# Loss of Crystalline Lens by Blow on Eyeball

**Published:** 1876-04

**Authors:** Thomas H. Kenan

**Affiliations:** 2d Assistant Physician, Georgia Lunatic Asylum


THE
### SOUTHERN MEDICAL RECORD.

VOL. VI. APRIL, 1876. NO. 4.

### Original Communications.

### LOSS OF CRYSTALLINE LENS, BY BLOW ON EYE BALL,

By THOMAS H. KENAN, 2d Assistant Physician, Georgia Lunatic Asylum.

Mr. J. B. Lunatic and Epileptic. Age 69. Fell and struck left eye against edge of steps leading from portico. Upon being called to him, I found the lens on the floor, and after securing it, examined the eye carefully and found the wound through the sclerotic coat very near the cornea on the nasal side, with slight loss of blood and aqueous humor. There being no rupture save the one made by the edge of the steps, the lens must have been forced out at that point by the resiliency of the vitreous body. He was put to bed and I gave him morphine, gr. ⅓, to be repeated during the night if necessary. Ordered a solution of arnica and laudanum to be applied to the parts. It was with great difficulty that he was kept in bed and the dressing applied, and in spite of every effort, he would not give his co-operation in the treatment. The following day there was some swelling of lids and ecchymosis, but the swelling soon subsided. There was but very little conjunctivitis and no trouble to speedy recovery.

The eye is now filled out. The pupil is elliptical, and there is a fold of the Iris, caught in the wound of sclerotic.

His bowels having been kept regular, and an occasional dose of hydrate of chloral is about all the treatment, save, of course, the water dressing, which was continued for 10 or 15 days, after the discontinuance of the solution of arnica, at the expiration of the first 48 hours. He is now up and about. General health as good as for the last year or two.

